# KEGG orthology prediction of bacterial proteins using natural language processing

**DOI:** 10.1186/s12859-024-05766-x

**Published:** 2024-04-11

**Authors:** Jing Chen, Haoyu Wu, Ning Wang

**Affiliations:** 1 School of Artificial Intelligence and Computer Science, Jiangnan University, Wuxi, China; 2 Jiangsu Provincial Engineering Laboratory of Pattern Recognition and Computing Intelligence, Jiangnan University, Wuxi, China

**Keywords:** KEGG orthology, Protein function prediction, Protein language model, Deep learning

## Abstract

**Background:**

The advent of high-throughput technologies has led to an exponential increase in uncharacterized bacterial protein sequences, surpassing the capacity of manual curation. A large number of bacterial protein sequences remain unannotated by Kyoto Encyclopedia of Genes and Genomes (KEGG) orthology, making it necessary to use auto annotation tools. These tools are now indispensable in the biological research landscape, bridging the gap between the vastness of unannotated sequences and meaningful biological insights.

**Results:**

In this work, we propose a novel pipeline for KEGG orthology annotation of bacterial protein sequences that uses natural language processing and deep learning. To assess the effectiveness of our pipeline, we conducted evaluations using the genomes of two randomly selected species from the KEGG database. In our evaluation, we obtain competitive results on precision, recall, and F1 score, with values of 0.948, 0.947, and 0.947, respectively.

**Conclusions:**

Our experimental results suggest that our pipeline demonstrates performance comparable to traditional methods and excels in identifying distant relatives with low sequence identity. This demonstrates the potential of our pipeline to significantly improve the accuracy and comprehensiveness of KEGG orthology annotation, thereby advancing our understanding of functional relationships within biological systems.

## Background

Bacteria, ubiquitous microorganisms inhabiting diverse environments, play an indispensable role in shaping the biosphere and influencing human health [1–3]. Their sheer abundance and diversity underscore their significance in ecological processes, ranging from nutrient cycling to bioremediation [4–6]. Moreover, bacteria have been central to pivotal discoveries in the fields of genetics, molecular biology, and biotechnology, serving as model organisms for fundamental biological research. The functional elucidation of bacterial proteins is pivotal in unraveling the intricacies of microbial life and harnessing their potential for biotechnological applications.

With the advent of high-throughput technologies, the number of newly discovered bacterial proteins per year is increasing rapidly [7]. While this wealth of genetic information offers immense potential for elucidating the roles and functions of these proteins, annotating the functions of newly discovered sequences remains a formidable challenge. Traditional experimental methods for function annotation, whether in vitro or in vivo, are not only expensive but also time-consuming. Consequently, there is an urgent need to explore alternative, cost-effective strategies for protein function prediction. One promising method is the application of automated annotation tools, which use computational methods to predict protein functions based on sequences.

These automated annotation tools rely on databases that have been manually curated and annotated by human experts. One widely used database for gene and protein functional annotation is the KEGG database [8]. It comprises comprehensive and integrated databases of molecular pathways, networks, and genes involved in various cellular processes, including metabolism, signaling, and diseases. The KEGG orthology (KO) database is a database of molecular functions represented in terms of functional orthologs. A functional ortholog is manually defined in the context of KEGG molecular networks. The KO identifier (called K number) is defined based on the experimental characterization of genes and proteins within specific organisms. These K numbers are subsequently used to assign orthologous genes in other organisms. KO data refers to the protein sequences cataloged within the KO database, whereas non-KO data references protein sequences identified in the KEGG GENES database yet to be associated with a KO identifier. Accurate and reliable KO prediction is essential for understanding the biological systems.

Several computational methods have been proposed for KO prediction, including sequence alignment and machine learning. KOBAS [9–11] used BLAST [12] E-value to assign K numbers. KAAS [13] employed BLAST to compute the bidirectional hit rate between query sequences and the KEGG reference databases. It defined a weighted score to assign K numbers, and these weighting factors take into account aspects such as ortholog group and sequence length, among others. BlastKOALA and GhostKOALA [14] used BLASTP and GHOSTX [15], respectively, for searching the non-redundant KEGG GENES database. KOALA (KEGG Orthology And Links Annotation) was originally developed as KEGG’s internal annotation tool for K number assignment using SSEARCH [16] computation. The scoring methodology of KOALA takes into account numerous factors. These include the Smith-Waterman (SW) score [17], the best-best flag, the degree of alignment overlap, the ratio of query to DB (DataBase) sequences, the taxonomic category, and the presence of Pfam domains. In BlastKOALA, the K number assignment is performed using the weighted sum of BLAST bit scores, where the weighting scheme is the same as the KOALA algorithm excluding the bidirectional best-hit information. In GhostKOALA, the K number assignment is simply based on the sum of GHOSTX normalized scores without considering any weighting factors. KofamKOALA [18] used profile hidden Markov models (pHMM) from machine learning to calculate similarity scores and subsequently also used the KOALA algorithm to assign K numbers.

KOBAS, KAAS, and BlastKOALA all utilize the BLAST algorithm to calculate sequence similarity but employ distinct methods for scoring computation. KOALA differentiates itself by incorporating additional information, such as taxonomic categories and Pfam domains, which often contribute to improved results. BlastKOALA and GhostKOALA, while both based on KOALA, adopt different approaches to sequence similarity calculation. BlastKOALA utilizes BLASTP, a heuristic local alignment algorithm, which is particularly suited for annotating fully sequenced genomes. On the other hand, GhostKOALA leverages GHOSTX, which employs genome-wide sequence alignment and uses suffix arrays for efficient matching. Unlike BLASTP, GHOSTX is designed for protein-level comparisons at the genomic scale, making it ideal for conducting comprehensive genome searches and homology analysis in large-scale genome data. KofamKOALA presents a different approach compared to BlastKOALA and GhostKOALA. It employs the KOALA framework but also integrates the use of a HMM profiles database for KEGG Orthologs, known as KOfam. This method allows KofamKOALA to provide accurate functional annotations by matching query sequences using HMM profiles instead of actual sequences. An additional advantage of KofamKOALA is its speed, as the use of HMM profiles can significantly speed up the matching process. However, note that after database updates, a substantial amount of time is needed to update these HMM profiles, which could be a potential limitation. Choosing between these methods largely depends on the specific characteristics of the dataset in question and the specific constraints of the study.

However, these methods have certain limitations, as they rely on sequence similarity and may not be effective in identifying KOs with dissimilar sequences. Around one-third of identified bacterial proteins lack known homologs, thereby restricting the number of annotations that can be accurately predicted [19]. Moreover, the growing reliance on high-throughput experiments has resulted in a skewed distribution of functional protein annotations in databases, leaving a considerable number of bacterial proteins unexplored in terms of their functions [20]. In recent years, deep learning has emerged as a promising method for protein function prediction, owing to its capacity to autonomously learn complex patterns and representations from large and complex datasets.

Anfinsen proposed the famous sequence-structure-function relationship in 1973 [21], which states that the protein sequence determines its structure, and the structure determines its function. Since the protein sequence is composed of amino acids and has a hierarchical structure similar to sentences and words, NLP (Natural Language Processing) can be used to model and learn protein sequences and predict protein functions. Compared to the previous sequence similarity-based methods, using NLP methods with deep learning for KO prediction can discover KOs that have similar functions but dissimilar sequences. These methods primarily involves extracting features from the protein sequence, converting them into word representations (embeddings), and subsequently classifying these representations. These methods can be classified into three categories: context-free models, context-sensitive models, and pre-trained large-scale protein language models. Context-free models generate a unique word representation for each amino acid(AA) [22, 23]. While context-sensitive models produce representations that depend on the context in which the AA appears [24, 25]. Therefore, a single AA may have different representations across different protein sequences. Pre-trained large-scale protein language models have extracted many biological features from protein sequences through unsupervised pre-training on a large corpus, and fine-tuning or feature extraction in downstream tasks can achieve good results [26–28]. In theory, embedding-based methods offer an alternative perspective for annotation, employing techniques such as clustering to overcome the limitations of homology-based methods.

In this paper, we propose a novel pipeline for the KO annotation of bacterial sequences using NLP and deep learning. *Firstly*, we propose a classifier based on pre-trained large-scale protein language models to distinguish between KO and non-KO data. *Subsequently*, an embeddings-based clustering module is conducted to assign a specific K number to each candidate sequence. *Furthermore*, we conduct a structural alignment method, using structural similarity, to ascertain the functional similarity of sequences, thus validating the assigned KOs. Our pipeline demonstrates competitive performance compared to traditional methods and notably excels in identifying distant relatives with low sequence identity. To the best of our knowledge, this study represents the pioneering effort in using a deep learning model that incorporates NLP for computational modeling in KO prediction.

## Results

### Overview of our pipeline for KO annotation

**Fig. 1:**
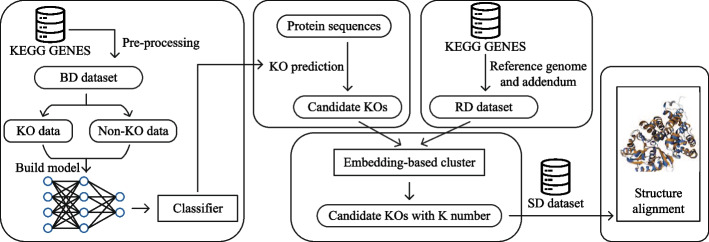
Schematic overview of our pipeline. In this study, we started by collecting KO and non-KO data from the KEGG GENES database to construct our classifier (left). Subsequently, we employed the classifier to mine protein sequences for the identification of potential KOs and used an embedding-based clustering module to assign a specific K number (middle). To validate our results, we performed structural alignment between the candidate KO sequences and the known sequences in the KEGG database (right)

In Fig. 1, we present a schematic overview of the proposed KO annotation pipeline. This pipeline is comprised of two primary parts: a classifier designed to discriminate between candidate KO sequences and non-KO sequences, and a clustering module that subsequently assigns a specific K number to each candidate KO sequence. To validate our results, we performed structural alignment between the candidate KO sequences and the known sequences in the KEGG database. To train the classifier, we used the BD (Bacterial Data) dataset, which consists of pre-processed bacterial protein sequences sourced from KEGG GENES, totaling approximately 17 million sequences. The cluster module used the RD (Reference Data) dataset, which comprises reference genomes and addendum from KEGG GENES, totaling approximately 0.6 million sequences. For comprehensive information regarding the construction of both the BD and RD datasets, please refer to Data collection and filtering for detailed explanations.

In order to provide a more tangible understanding of our pipeline, we present a running example. Let’s consider an unannotated sequence, which matches with the annotated sequence ppu:PP_4955, associated with the KEGG number K02030. The process can be broken down into the following steps:

1. Sequence Embedding: The unannotated sequence is first transformed into an embedding using ProtT5. This sequence embedding captures the essential features of the sequence.
2. KO Prediction: This sequence embedding is subsequently input into a Multilayer Perceptron (MLP) layer, which acts as our primary prediction model. The MLP layer is used to predict whether the sequence is a KO or not, determining if the process proceeds to the clustering step or terminates.
3. Sequence Clustering: For sequences predicted as KO, the sequence embedding is compared to the embeddings of each sequence in the RD dataset. This comparison is performed using Euclidean distance as the similarity metric.
4. Annotation Assignment: The sequence (in this case, ppu:PP_4955) that exhibits the smallest Euclidean distance is chosen as the best match. The annotation associated with this best match (K02030 in our example) is then assigned to the initially unannotated sequence.

### Performance evaluation of classifiers

Table 1 presents a comprehensive summary of the performance metrics obtained by evaluating various classifiers. The training dataset comprised 80% of the BD database, while the remaining 20% was allocated for testing purposes. The evaluated metrics encompass precision, recall, and the F1 score, which represents the harmonic mean of precision and recall. The LSTM (Long Short-Term Memory) model was based on Veltri et al. [29], which is a neural network model with a core layer of LSTM [30]. The attention model was inspired by Ma et al. [31], where the LSTM layer was replaced with an attention layer [32]. Finally, we included a Text-to-Text Transfer Transformer (ProtT5) model that was pre-trained using a large number of protein sequences. Notably, the ProtT5 model outperforms all other classifiers across all metrics, showcasing its superior predictive capabilities for KO annotation. With compelling results displayed in Table 1, we confidently select the ProtT5 model as the preferred classifier for our study.

**Table 1:** Performance comparison with different classifiers

Model	$\text {Precision}^*$	$\text {Recall}^*$	$\text {F1}^*$
LSTM	0.899	0.870	0.884
Attention	0.795	0.798	0.797
ProtT5	**0.960**	**0.967**	**0.963**

Best performance is marked in bold. The ProtT5 model exhibits superior performance across all evaluation metrics

In order to distinguish the experimental results of the classifier from the entire pipeline, precision, recall, and F1 here are marked with asterisks as superscripts

### Performance evaluation of KO annotation tools

To validate the results, we implemented a evaluation that involved the random selection of two species, Bradyrhizobium japonicum E109 (bjp) and Paraburkholderia aromaticivorans BN5 (parb), from the KEGG organisms. A test set comprising 12,329 sequences from these selected species was used to evaluate the performance of each KO annotation tool. The test set had a ratio of 1.09:1 for KO to no KO assigned sequences. Sequences from the BD dataset that were identical to those from the two species were removed, leaving the remaining sequences as the training set for the classifier. In cases where identical sequences from different species exhibited varying annotations, we retained the annotation with the K number as the final annotation.

Our clustering module still relies on the RD dataset, which does not include sequences from these two species. As for BlastKOALA, GhostKOALA, and KofamKOALA, we used the default target databases of their respective webservers. Our RD dataset is largely consistent with the dataset used by BlastKOALA, while GhostKOALA employed a dataset that is one order of magnitude larger. KofamKOALA, on the other hand, utilized 25,346 pHMMs.

The evaluation of each tool contains the computation of the number of match, unmatch, missed, and added cases, alongside precision, recall, and F1 score calculations. Specifically, match refers to the number of cases where the predicted KO precisely matched the KO defined in the KEGG GENES database. Unmatch denotes cases where the predicted KO differed from the assigned KO in KEGG GENES. Missed cases represented KOs defined in KEGG GENES that were not successfully predicted by the tools. Finally, added cases indicated situations where a K number was assigned by the prediction despite no corresponding KO being defined in KEGG GENES.

In Table 2, our pipeline achieved the best recall by having the highest number of match cases, the lowest number of missed cases, and the second-best F1 score. GhostKOALA obtained the best precision and F1 score due to having the fewest unmatch cases. And BlastKOALA had the lowest number of added cases. GhostKOALA’s precision is relatively higher, owing to the larger dataset, which has the potential to improve the accuracy of predictions. Due to the differences in datasets, our pipeline’s performance evaluation with BlastKOALA is the most equitable. Our pipeline outperforms BlastKOALA with higher match cases, recall, and F1 scores.

**Table 2:** Performance comparison with other KO annotation tools

Method	Match	Unmatch	Missed	Added	Precision	Recall	F1
BlastKOALA	6172	64	552	**100**	0.974	0.909	0.941
GhostKOALA	6423	**26**	339	117	**0.978**	0.946	**0.962**
KofamKOALA	5955	88	745	953	0.851	0.877	0.864
Ours	**6426**	183	**179**	171	0.948	**0.947**	0.947
Ours w/o classifier	5943	62	783	407	0.876	0.927	0.900
Ours with threshold	6399	169	220	151	0.952	0.943	0.948

Best performance is marked in bold. We calculated the number of match (predicted KO is identical to the KO defined in KEGG GENES), unmatch (predicted KO is different from the KO defined in KEGG GENES), missed (the KO is defined in KEGG GENES but no prediction was made), and added (no KO is defined in KEGG GENES, but the prediction assigned a K number) for each tool, along with precision, recall, and F1 score

If classifier is not used, and a clustering threshold is employed to distinguish between KO and non-KO sequences, the metrics show inferior performance compared to the original pipeline that just used classifier. It indicates that relying solely on a clustering threshold may not capture the complexity and nuances required for accurate KO prediction. On the other hand, when both the classifier and clustering threshold are used simultaneously to differentiate KO and non-KO sequences, precision increases while recall decreases. However, the F1 score, which considers both precision and recall, remains almost the same. It suggests that the integration of classifier and clustering threshold allows for a more refined and precise classification of sequences. It is important to note that in this study, a threshold-based method was not utilized to avoid introducing excessive hyperparameters.

### Generalizability across different bacterial species

As a critical measure of the robustness and utility of a model is its ability to generalize across diverse datasets, we extended the evaluation to assess our pipeline’s performance across different bacterial species. Initially, Bradyrhizobium japonicum E109 (bjp) and Paraburkholderia aromaticivorans BN5 (parb), were randomly selected from the KEGG database. These species, belonging to the Alphaproteobacteria and Betaproteobacteria classes within the Pseudomonadota phylum respectively. To augment our pipeline’s generalizability assessment, we randomly selected a bacterial species from a different phylum in the KEGG database, added after our initial download. We ultimately chose Borreliella finlandensis Z11 (bff) from the phylum Spirochaetota. The performance metrics of our pipeline on this additional species were congruent with our initial results, further substantiating our pipeline’s generalization potential. The performance results are listed in Table 3.

**Table 3:** Performance metrics of our pipeline across different bacterial species

Species	Precision	Recall	F1
bjp	0.946	0.946	0.946
parb	0.949	0.947	0.948
bff	0.946	0.982	0.964

This table presents the precision, recall, and F1 score for each of the three bacterial species evaluated: Bradyrhizobium japonicum E109 (bjp), Paraburkholderia aromaticivorans BN5 (parb), and Borreliella finlandensis Z11 (bff)

### Validating results through structural alignment

**Fig. 2:**
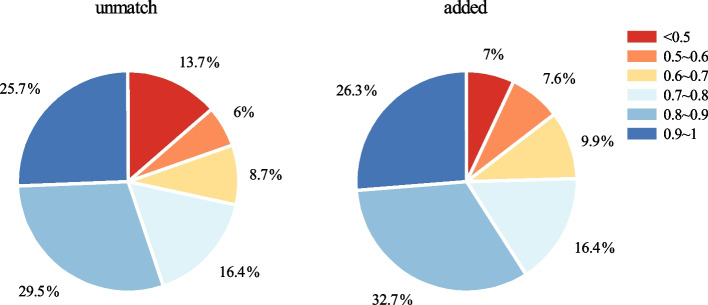
Distribution of structural similarity metric TM-score in unmatch and added cases. These two cases represent instances where our pipeline incorrectly assigned the K number, while the KEGG GENES database assigned a different K number (unmatch) or did not assign K number (added). A TM-score of $\ge 0.5$ suggests the presence of similar structural domains, while a TM-score of $\ge 0.8$ indicates highly similar structures, which implies potential functional similarity

To evaluate the functional similarity in the unmatch and added cases, we conducted structural alignments between the known KO sequence and the KO sequence identified by our pipeline using the CE-CP (Combinatorial Extension for Circular Permutations) algorithm [33]. The quality of these alignments was assessed using the TM-score (Template Modeling score) [34], a score between (0, 1], where 1 indicates a perfect match between two structures. Therefore, a higher TM-score reflects a greater level of structural similarity. The results of these structural alignments are shown in Fig. 2. In the unmatch cases, where the assigned K number differ from those defined in KEGG GENES, we found that 55.2% of the sequences had a TM-score $\ge 0.8$, indicating a high level of structural similarity. Only 13.7% of the sequences had a TM-score <0.5, suggesting dissimilar structural domains. Similarly, in the added cases, where our pipeline assigned the K number to sequences not defined in KEGG GENES, we observed that 59% of the sequences had a TM-score $\ge 0.8$, while only 7% had a TM-score <0.5. Within the unmatch cases, we found that 13.7% of the sequences had different KO numbers but belonged to the same EC number, suggesting shared enzymatic functions. For example, for the sequence parb:CJU94_35085, we assign K10010, whereas KEGG assigns K02028, but they share the same EC:7.4.2.1. And the TM-score between the parb:CJU94_35085 and our clustered sequence is 0.99. The findings indicate that despite differences in the assigned K number, the functionalities of the sequences are quite similar due to the high structural similarity.

### Exploring recognition of distant relatives

**Fig. 3:**
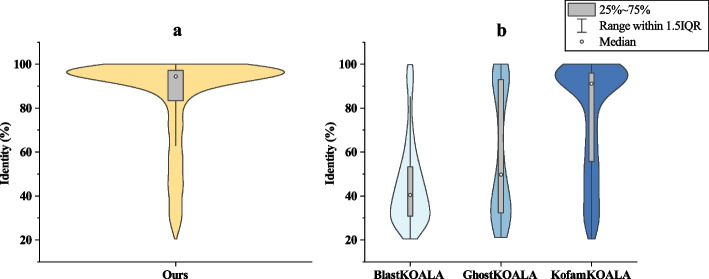
Identity distribution. The width of the violin plot along the X-axis corresponds to the frequency of data points. **a** The identity distribution of the predicted sequences of all match cases and the clustered sequence. **b** The identity distribution of the sequences not predicted by other methods in our match cases

Our pipeline achieved the highest number of match cases, prompting us to conduct further analysis. We used the Smith-Waterman algorithm [35] to compute the identity between the predicted sequences of all match cases and the clustered sequence, as shown in Fig. 3a. Additionally, we calculated the identity of the sequences not predicted by other methods in our match cases, as shown in Fig. 3b.

Based on the analysis of Fig. 3, the identity distribution of sequences in our match cases mostly falls within the range of 80% or higher. However, for sequences not predicted by BlastKOALA and GhostKOALA in our match cases, the majority of identities are in the 60% or lower range. This indicates that our model has a stronger ability to identify distant relative proteins, despite GhostKOALA’s using a dataset that is one order of magnitude larger than ours. KofamKOALA displays a similar overall trend to our model, but it identifies fewer match cases compared to ours.

We provided two low identity (< 30%) sequences from our match cases as examples where other methods failed to make predictions. Sequence parb:CJU94_35185 exhibits only 21.2% identity with the clustered sequence eba:p2A55, yet they are remarkably close in the embedding space, allowing our model to recognize it. Likewise, another sequence bjp:RN69_21090 and the clustered sequence ppu:PP_4955, showcase a sequence identity of 24.3%, but close in the embedding space.

## Discussion

Annotating bacterial proteins with KO classifications is crucial for deciphering the functional roles of these proteins within the intricate machinery of microbial organisms. The comprehensive understanding of these annotations aids in elucidating the pathways, metabolic networks, and regulatory mechanisms that govern bacterial life. Accurate KO annotations are pivotal for various downstream analyses, including comparative genomics, pathway reconstruction, and functional inference.

In this study, we present a novel pipeline for predicting KO annotations of bacterial proteins using NLP from deep learning. Our model’s performance surpasses most traditional methods, falling slightly short only in comparison to GhostKOALA. However, it is important to note that GhostKOALA operates on a dataset that is an order of magnitude larger, which may account for the nuanced differences in performance. On the other hand, BlastKOALA uses a dataset that is largely consistent with our RD dataset, and our pipeline outperforms BlastKOALA with superior match cases, recall, and F1 scores.

In the comparison of the performance of three classifiers, the ProtT5 model outperforms the other two classifiers across all metrics. The ProtT5 model was pre-trained using approximately 45 million protein sequences, with the pre-training task involving learning to predict masked amino acids (tokens) within known sequences. Subsequently, it trained on our BD dataset with 17 million bacterial protein sequences using MLP to distinguish between KO and non-KO sequences. In contrast to LSTM and attention models trained solely on the BD dataset without pre-training, the extensive pre-training on a large dataset enabled ProtT5 to acquire a deeper understanding of the intricate language of life. This understanding contributed to its superior performance in our classification tasks.

We explored the use of both classifiers and clustering thresholds. Our findings indicate that employing classifiers, particularly those generated using pre-trained models to generate embeddings, offers a more effective method compared to solely relying on clustering thresholds. Combining the classifier and clustering thresholds allows for finer adjustments, enabling researchers to prioritize precision or recall depending on the specific needs of their analysis.

To further validate the accuracy of our predictions for the sequences in our unmatch and added cases, we conducted structural alignments. Although we did not precisely predict the matching K number, approximately 89.7% of the sequences exhibited TM-score greater than 0.5. This suggests that these proteins share similar structural domains and likely perform analogous functions. In the case of unmatch, 13.7% of the sequences possessed different KO assignments but shared identical EC numbers, indicating shared enzymatic function.

One of the most significant challenges in annotating bacterial proteins lies in the ability to capture functional relationships between proteins that share low sequence similarity. Traditional methods predominantly rely on sequence homology, which can overlook crucial associations, particularly among distantly related proteins. Our analysis revealed that a proportion of the KO proteins our model identified were missed by traditional methods, particularly those with low sequence similarity. This suggests that our NLP-based pipeline has the potential to uncover functional relationships that may be obscured by conventional homology-based methods.

While methods such as I-TASSER [36], which are based on protein 3D structures, may mitigate an over-reliance on sequence similarity alone, they often need significant computational resources and time. To illustrate, generating a protein structure with 384 residues using a V100 GPU card with 16GB memory can take approximately 9.2 min. This can be quite resource-intensive when dealing with large datasets. In contrast, our pipeline is far more efficient. More specifically, generating embeddings for a protein of the same length using the same GPU card takes only 0.057 s. Further, our study explores the feasibility and effectiveness of using embeddings from a pre-trained large-scale protein language model, solely based on sequence information, for functional clustering. We have also cross-validated our results using AlphaFold2, which demonstrated satisfactory performance. This approach, while being economical and efficient, also proves to be accurate, offering a viable alternative for KO prediction.

Despite the innovative approach and encouraging results achieved by our method, it is important to recognize certain limitations. Firstly, the substantial computational resources demanded by the large protein language model ProtT5 present a challenge. Specifically, the ability to process long sequences is constrained by the memory capacity of the GPU used. This requirement thus restricts the range of sequence lengths that our method can effectively handle. Furthermore, our pipeline currently focuses on sequence data. Despite its ability to yield important information, this approach might not comprehensively capture the intricate characteristics of proteins. This focus on sequences could potentially leave out important information derived from other protein characteristics, such as their three-dimensional structures or interactions within biological systems.

By effectively identifying and annotating new or unknown bacterial proteins, our pipeline contributes to an increased annotation coverage of bacterial proteins in the KEGG database, thereby expanding its application scope. Furthermore, the integration of our pipeline with NLP technologies offers a fresh perspective and methodology for future research in the KO prediction domain. It can be effectively applied to other species and extended to other protein function predictions, further amplifying its utility and impact.

## Conclusions

This study introduces a novel NLP-based pipeline to the field of KO prediction and demonstrates its significant potential. Our pipeline excels in predicting distant relatives, providing a new solution to address the challenges faced by traditional homology-based methods.

For future research, we suggest exploring the integration of NLP-based methods with traditional methods to fully use their complementary advantages in KO prediction, thus improving prediction accuracy and comprehensiveness. In KEGG GENES, approximately 20% of bacterial protein sequences have a length greater than 600. Therefore, another direction is the analysis of long Transformers, which can handle longer amino acid sequences without preprocessing steps and significant computational resources. As a final point, we consider incorporating other features, such as KEGG pathways (molecular interaction, reaction, and relation networks) and protein structure information, to further enhance the performance of model.

## Methods

### Data collection and filtering

We collected three datasets, including BD, RD, and SD (Structural Data) datasets.

**Fig. 4:**
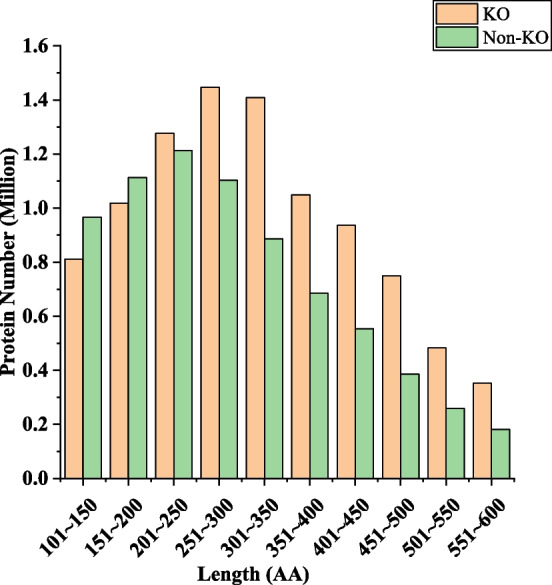
Length distribution of BD dataset

The BD dataset contains both KO and non-KO data, which were obtained from the KEGG GENES database (downloaded in August 2022) with a restriction on the species to bacteria (7409 species in total in KEGG GENES). Duplicate sequences were removed. To ensure the quality of the data, we removed sequences shorter than 100 amino acids and sequences longer than 600 amino acids. We set these size limits based on the observation that sequences shorter than 100 amino acids often have lower true positives [37], while sequences longer than 600 amino acids contain limited KO data (less than 20%). Sequences containing undefined amino acids were also removed (0.07%). The length distributions of KO and non-KO data were kept consistent (deviation <5%) to avoid length bias in the model. The data were split into training and testing sets with an 8:2 ratio, and data from all species were merged. In cases where identical sequences from different species exhibited varying annotations, we retained the annotation with the K number as the final annotation. The final training set consisted of 7,624,360 KO sequences and 1,906,089 non-KO sequences, while the testing set consisted of 5,875,496 KO sequences and 1,468,873 non-KO sequences. The length distribution is shown in Fig. 4.

The RD dataset is a small subset of KEGG GENES containing KEGG reference genomes and individual sequences linked from PubMed records of KO entries. Reference genomes are introduced for those genomes with enough experimental data for gene/protein functions, as seen by the number of sequence links in the PubMed reference fields of the KO database. We obtained 24,146 KOs and 623,239 reference sequences (Table 4).

**Table 4:** Information about datasets

Data name	BD	RD
Number of proteins (in k)	16,874	623
Number of amino acids (in m)	5121	296
Disk space (in MB)	6550	335

Units: number of proteins in thousands (k), of amino acids in millions (m), and of disk space in MB (uncompressed storage as text)

The SD dataset contains protein structures. The structures of predicted sequences are generated using AlphaFold2 [38], while the structures of KEGG sequences are obtained from Protein Data Bank (PDB) [39] or AlphaFold Protein Structure Database (AFDB). Although AlphaFold2 is a predictive model, it has been shown to achieve atomic-level precision that is comparable to experimental protein structure resolution [38]. Therefore, structures generated by AlphaFold2 are considered to have high confidence.

### Classifier

We trained three models to distinguish KOs from non-KOs. LSTM and Attention did not use pre-training. ProtT5 [28] used pre-training on biological language corpora.

**Fig. 5:**
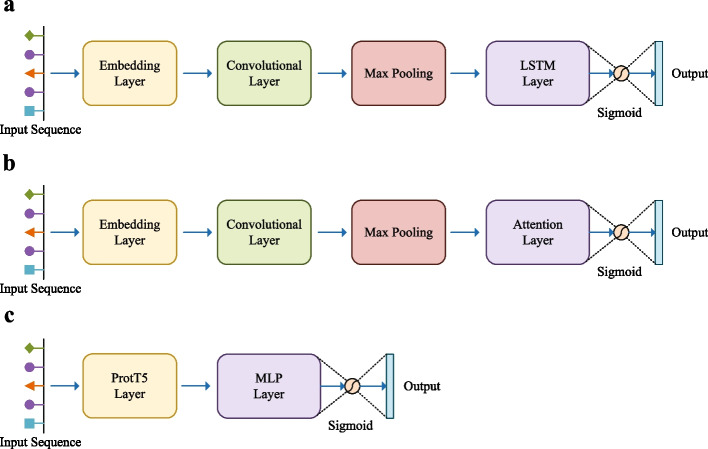
Classifier architecture. **a** The LSTM model architecture. The protein sequences were converted into fixed-size vectors and subsequently passed through an embedding layer with a length of 128. This was followed by a 1D convolutional layer comprising 64 filters and a subsequent 1D max pooling layer. Next, an LSTM layer with 100 units was implemented, followed by a final classification layer that employed a sigmoid function. **b** The attention model architecture. The attention model replaced the LSTM layer of the LSTM model with an attention layer, while the remaining modules remained unchanged. **c** The ProtT5 model architecture. The protein sequences were initially fed into the ProtT5 Layer, followed by an MLP Layer comprised of two fully connected layers with a hidden size of 100. Just like the LSTM and attention method, the final step used a sigmoid function for classification

The LSTM model originates from the research conducted by Veltri et al. [29], while the Attention model is also based on earlier research by Ma et al. [31]. Firstly, we converted the protein sequences into fixed-size vectors by representing the 20 basic amino acids as numerical values ranging from 1 to 20. If the raw sequence did not reach 600 amino acids, we padded the sequence vectors with 0. The resulting vector was then expanded to 128 dimensions using an embedding layer, and fed into a 1D convolutional layer with 64 filters and a 1D max pooling layer. Secondly, an LSTM layer with 100 units was implemented, followed by a final classification layer that employed a sigmoid function (Fig. 5a). The attention model simply replaces the LSTM layer with an attention layer, while keeping the rest of the network unchanged (Fig. 5b).

ProtT5 (ProtT5-XL-U50) is trained on a large corpus of protein sequences. This allows it to learn representations that are particularly well-suited for protein-related tasks, such as predicting protein structure, function, and interactions. By feeding protein sequences into the model and extracting the last hidden layer representations generated by the model, we can obtain high-quality, low-dimensional representations of proteins that can be used as input to downstream models [40]. For our downstream model, we used an MLP architecture consisting of two fully connected layers with a hidden size of 100. The final classification was performed using the sigmoid activation function (Fig. 5c).

Binary cross-entropy loss, Adam optimizer [41], and the ReLU activation function were selected for all models. To prevent overfitting, we reserved 20% of the training dataset as the validation dataset, which was employed to implement the early-stop strategy. The strategy halted the model’s training when its performance began to decline, and the best-performing model on the validation dataset was saved as the final model. The final classification layer produced a scalar value between 0 and 1, with values greater than 0.5 classified as KO.

To evaluate and compare the three classifiers, we used three evaluation metrics: $\text {precision}^*$, $\text {recall}^*$, and $\text {F1}^*$. To distinguish the calculation formulas for precision and recall of the classifier from the entire pipeline, an asterisk (*) is added as a superscript here. $\text {Precision}^*$ measures the proportion of true positives out of all predicted positives, while $\text {recall}^*$ measures the proportion of true positives out of all actual positives. Since $\text {precision}^*$ and $\text {recall}^*$ can sometimes conflict with each other, a common way to combine them is through the $\text {F1}^*$ score, which is the harmonic mean of $\text {precision}^*$ and $\text {recall}^*$. The $\text {F1}^*$ score provides a balanced measure of model performance that takes both $\text {precision}^*$ and $\text {recall}^*$ into account, and is therefore often used as an overall indicator of a model’s classification ability. The definition of the formula is as follows:

1 $$\begin{aligned} \text {Precision}^*&= \frac{\text{TP}}{\text{TP} + \text{FP}} \end{aligned}$$

2 $$\begin{aligned} \text {Recall}^*&= \frac{\text{TP}}{\text{TP} + \text{FN}} \end{aligned}$$

3 $$\begin{aligned} \text {F1}^*&= \frac{2}{\mathrm {(Recall^*)}^{-1} + \mathrm {(Precision^*)}^{-1}} \end{aligned}$$

where TP (True Positive) represents the number of real positive cases where the model correctly predicted a positive result, FP (False Positive) represents the number of real negative cases where the model incorrectly predicted a positive result, and FN (False Negative) represents the number of real positive cases where the model incorrectly predicted a negative result.

### Clustering

The process of clustering predicted KOs and known KOs from the RD dataset based on similar functions begins with the conversion of protein sequences into embeddings using ProtT5. Subsequently, the Euclidean distance (calculated using Eq. (4)) is calculated between the embeddings of the predicted sequences and those of the known KOs. The best match is selected based on the smallest Euclidean distance, and the associated annotation of the best match is subsequently assigned to the predicted sequence. In cases where there are several top matches with different annotated K number, our pipeline is designed to report all such matches. While theoretically it’s possible to have multiple top matches, the likelihood of is extremely low due to the high dimensionality and complexity of protein embeddings. Thus, in our experiments, we have not encountered such cases.

4 $$\begin{aligned} d(x,y)=\sqrt{(x_1-y_1)^2 +(x_2-y_2)^2 + \cdots +(x_n-y_n)^2} \end{aligned}$$

where $x=(x_1,\ldots ,x_n)$ and $y=(y_1,\ldots ,y_n)$ are n-dimensional embeddings of two protein sequences.

We selected the ProtT5 model to convert the protein sequence into embeddings due to its superior performance, as observed in the experimental results of ProtTrans [28] and our classifier experiment. Among the models evaluated, ProtT5 exhibited the most comprehensive and effective performance, making it the preferred choice for generating embeddings from protein sequences in our study.

For a comprehensive evaluation, we use precision, recall, and F1 score. While precision and recall bear similarities to those used in classification, there exist subtle distinctions. Specific details can be found in Eqs. (5) and (6).

5 $$\begin{aligned} \text {Precision}&= \frac{\text{match}}{\text{match}+\text{unmatch}+\text{added}} \end{aligned}$$

6 $$\begin{aligned} \text {Recall}&= \frac{\text{match}}{\text{match}+\text{unmatch}+\text{missed}} \end{aligned}$$

7 $$\begin{aligned} \text {F1}&= \frac{2}{\textrm{Recall}^{-1} + \textrm{Precision}^{-1}} \end{aligned}$$

Match refers to the number of cases where the predicted KO precisely matched the KO defined in the KEGG GENES database. Unmatch denotes cases where the predicted KO differed from the assigned KO in KEGG GENES. Missed cases represented KOs defined in KEGG GENES that were not successfully predicted by the tools. Added cases indicated situations where a K number was assigned by the prediction despite no corresponding KO being defined in KEGG GENES.

### Structural alignment

**Fig. 6:**
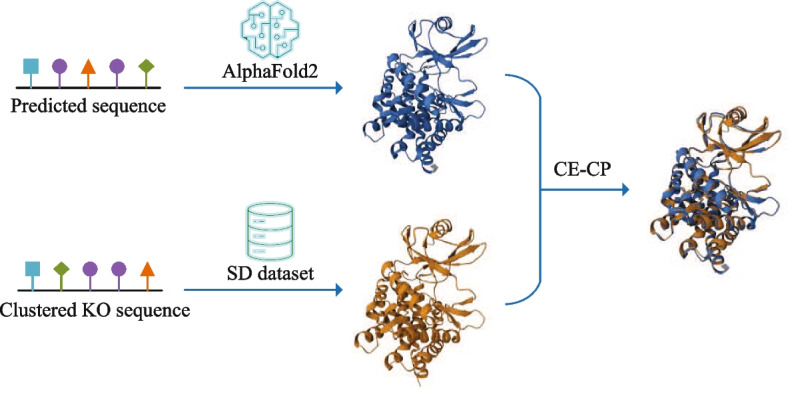
Structural alignment. The structure of the predicted sequence was generated using the AlphaFold2 model, while the structure of the clustered KO sequence was sourced from the PDB or AFDB databases. Structural alignment was conducted using the CE-CP algorithm

The predicted sequences are subjected to structural modeling using the highly precise AlphaFold2 model, renowned for its accuracy in protein structure prediction. Subsequently, these predicted structures are compared to the structures of clustered KO sequences, which are included in the SD dataset, using the CE-CP algorithm (Fig. 6). The CE-CP algorithm facilitates the comparison of circularly permuted proteins, enabling a comprehensive analysis of the structural similarities between the predicted sequences and the clustered KO sequences. We employed AlphaFold v2.3.2 with specific parameters configured as follows: model type: alphafold2_ptm, number relax: 0, templete mode: pdb70, msa mode: mmseqs2_uniref_env, pair mode: unpaired_paired, num recycles: 20, recycle early stop tolerance: tol = 0.5, max msa: auto, num seeds: 1, use dropout: False. For the CE-CP algorithm, specific parameters were set as follows: maximum gap size: 30, gap opening penalty: 5, gap extension penalty: 0.5, fragment size: 8, RMSD (Root Mean Square Deviation) threshold: 3, maximum RMSD: 99, and min CP block length: 5.

For evaluating structural comparison, the TM-score is used as the assessment metric. TM-score measures the proportion of the distance difference between matched residues in the target protein and template protein to the length of the target protein. The TM-score equation is presented in Eq. (8).

8 $$\begin{aligned} \text {TM-score}=\max \left[ \frac{1}{L_\text {target}}\sum _{i=1}^{L_\text {common}}\frac{1}{1+\left( \frac{d_i}{d_0(L_\text {target})}\right) ^2} \right] \end{aligned}$$

where $L_\text {target}$ is the length of the amino acid sequence of the target protein, and $L_\text {common}$ is the number of residues that appear in both the template and target structures.$d_i$ is the distance between the *i*th pair of residues in the template and target structures, and $d_0(L_\text {target})=1.24\root 3 \of {L_\text {target}-15}-1.8$ is a distance scale that normalizes distances.

## Data Availability

All code employed in this study is publicly available on GitHub (https://github.com/wuhaoyu3/KO-Identification). Publicly available datasets were analyzed in this study. These datasets were collected from the KEGG database (https://www.kegg.jp/), the PDB database (https://www.rcsb.org/), and the AFDB database (https://alphafold.ebi.ac.uk/).

## Abbreviations

KEGG: Kyoto encyclopedia of genes and genomes
KO: KEGG orthology
NLP: Natural language processing
AA: Amino acid
CE-CP: Combinatorial extension for circular permutations
TM-score: Template modeling score
PDB: Protein data bank
AFDB: AlphaFold protein structure database
BD: Bacterial data
RD: Reference data
SD: Structural data
DB: DataBase
MLP: Multilayer perceptron
HMM: Hidden Markov model
LSTM: Long short-term memory
RMSD: Root mean square deviation
TP: True positives
FP: False positives
FN: False negatives
